# Identification and Analysis of the AP2 Subfamily Transcription Factors in the Pecan (*Carya illinoinensis*)

**DOI:** 10.3390/ijms222413568

**Published:** 2021-12-17

**Authors:** Zhengfu Yang, Hongmiao Jin, Junhao Chen, Caiyun Li, Jiani Wang, Jie Luo, Zhengjia Wang

**Affiliations:** State Key Laboratory of Subtropical Silviculture, Zhejiang A&F University, Lin’an District, Hangzhou 311300, China; zafuyzf@163.com (Z.Y.); jhm@stu.zafu.edu.cn (H.J.); jlocke@163.com (J.C.); licaiyun@stu.zafu.edu.cn (C.L.); wangjiani@stu.zafu.edu.cn (J.W.); luojie0103@Outlook.com (J.L.)

**Keywords:** pecan, AP2 family, transcription factor, *CiANT5*

## Abstract

The AP2 transcriptional factors (TFs) belong to the APETALA2/ ethylene-responsive factor (AP2/ERF) superfamily and regulate various biological processes of plant growth and development, as well as response to biotic and abiotic stresses. However, genome-wide research on the AP2 subfamily TFs in the pecan (*Carya illinoinensis*) is rarely reported. In this paper, we identify 30 AP2 subfamily genes from pecans through a genome-wide search, and they were unevenly distributed on the pecan chromosomes. Then, a phylogenetic tree, gene structure and conserved motifs were further analyzed. The 30 AP2 genes were divided into euAP2, euANT and basalANT three clades. Moreover, the *cis*-acting elements analysis showed many light responsive elements, plant hormone-responsive elements and abiotic stress responsive elements are found in *CiAP2* promoters. Furthermore, a qPCR analysis showed that genes clustered together usually shared similar expression patterns in euAP2 and basalANT clades, while the expression pattern in the euANT clade varied greatly. In developing pecan fruits, *CiAP2-5*, *CiANT1* and *CiANT2* shared similar expression patterns, and their expression levels decreased with fruit development. *CiANT5* displayed the highest expression levels in developing fruits. The subcellular localization and transcriptional activation activity assay demonstrated that CiANT5 is located in the nucleus and functions as a transcription factor with transcriptional activation activity. These results help to comprehensively understand the pecan AP2 subfamily TFs and lay the foundation for further functional research on pecan AP2 family genes.

## 1. Introduction

The APETALA2/ethylene-responsive factor (AP2/ERF) is a superfamily of transcription factors (TFs), which were characterized by conserved AP2 domains. AP2/ERF superfamily TFs are mainly divided into the ERF (ethylene-responsive-element-binding protein), DREB (dehydration responsive element binding), AP2 (APETALA2), RAV (Related to ABI3/VP) and Soloists five major subfamilies [1,2]. Both the ERF and DREB subfamily members only contain one AP2 domain, and both are further subdivided into six subgroups [3]. The AP2 subfamily members containing two consecutive AP2 domains are further subdivided into euAP2, basalANT and euANT clades depending on the nuclear localization and amino acid sequences as well as other conserved motifs [4]. The RAV subfamily members contain a single AP2 domain and a B3 DNA-binding domain [5]. Although the Soloist subfamily with a single AP2 domain is displayed in most plants, its sequence and structure distinguish it from other AP2/ERF superfamily TFs [2,3]. The AP2/ERF domain consisted of a 60–70 amino acid functioning DNA binding domain; however, each of its subfamily recognized specific *cis*-acting elements. For example, ERF subfamily members usually bind to GCC-box responding to ethylene, pathogens and wounding [6], while DREB subfamily TFs usually bind to the DRE/CRT(A/GCCGAC) cis-element to activate drought and salt responsive genes [7]. The second AP2 domain in the AP2 subfamily binding to T/A-rich elements positively regulates floral organ identity [8], while in RAV1 it binds to the CAACA motif involved in ABA signaling [9,10]. Taken together, the diversification of gene structure and DNA-binding affinities may contribute to the different functions of the AP2/ERF superfamily members.

The AP2/ERF superfamily members have been reported to serve as regulators of stress responses and development processes, especially in reproductive organ development. *APETALA2* (*AP2*), expressed in floral organs and developing ovules as well as nonfloral organs, was initially identified as controlling flower and seed development in *Arabidopsis* [11]. Further research revealed that AP2 could bind to the AT-rich target sequence of *AGAMOUS* (*AG*) and directly activate its expression through the second AP2 domain [8]. *AINTEGUMENTA* (*ANT*), which belongs to the euANT clade, regulated cell division and organ size. The ectopic expression of *ANT* enlarged embryonic and all shoot organs by increasing cell numbers in *Arabidopsis* [12,13]. Moreover, seven high sequence similarity genes, *AINTEGUMENTA-like* (*AIL*), were identified, and they primarily expressed in young actively dividing tissues. The expression analysis by in situ hybridization demonstrated that *AIL5*, *AIL6* and *AIL7* expressed distinct spatial and temporal patterns in inflorescence meristems and flowers [14]. The overexpression of *AIL5* resulted in similar large floral patterns with ectopic expressions of *ANT*, while *AIL6*’s functioned redundancy with *ANT* took place during flower development [14,15]. In rice, the AP2 factor gene *RSR1* was identified as a negative regulator of type I starch syntheses genes, and the loss of function of *RSR1* resulted in an altered amylopectin structure and increased gelatinization temperature [16]. Interestingly, the same genetic locus was identified as the candidate of major QTL *qHD5*, controlling the heading date in rice [17]. *FRIZZY PANICLE* (*FZP*) was required to establish rice floral meristem identity and determine grain size [18,19]. *SUPERNUMERARY BRACT* (*SNB*) encoding *APETALA2-like* TF controlled rice seed shattering and seed size. A point mutation in *SNB* resulted in alternative splicing and reduced shattering in its mutant lines [20]. *EXCESSIVE NUMBEROF FLORAL ORGANS* (*ENO*) was identified as a tomato fruit regulator and regulating *WUSCHEL* expression. The disruption of *ENO* expanded the expression domain of *SlWUS* in a flower-specific manner, resulting in enlarged fruit size [21]. These studies demonstrated that the AP2 family TFs participated in the regulation of development processes, such as flower development, heading date, spikelet development and seed development.

With advances in sequencing technology, whole genomic sequences of more plants are available. The AP2 family genes were genome-wide identified and analyzed in increasingly more plant species, such as barley [22], common wheat [23] and *Dendrobium officinale* [24], which contributed to the understanding of the molecular function of AP2 family genes. However, the AP2 family TFs have not been reported in pecan (*Carya illinoinensis* (Wangenh.) K. Koch), which is considered a famous nut and woody oil tree species rich in unsaturated fatty acids [25]. In this study, we identify 30 AP2 subfamily TFs from pecans through a genome-wide search and mapped them on pecan chromosomes. Then, we construct a phylogenetic tree, analyzed gene structure and conserved motifs as well as promoter *cis*-acting elements. Furthermore, a qPCR is conducted to analyze their expression pattern, then subcellular localization and transcriptional activation activity are investigated. These results enrich our understanding of the AP2 family in pecans and lay the foundation for further functional research on pecan AP2 family genes.

## 2. Results

### 2.1. Identification of AP2 Subfamily Genes in the Pecan

**Figure 1:**
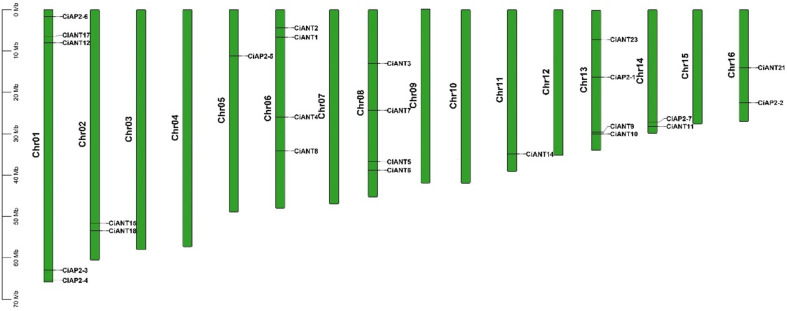
The distribution of the AP2 subfamily genes on pecan chromosomes. The position of each *CiAP2* was mapped according to its physical position on the pecan genome. The chromosome number is labeled at the right of each chromosome, and the scale is in mega bases (Mb).

To identify AP2/ERF family genes, an HMM search was employed to ascertain the genes containing the AP2 domain (Pfam ID:PF00847) from rice, *Arabidopsis* and pecan protein datasets. Then, 207 putative genes were retrieved from the pecan, and an evolutionary tree of AP2 family genes was initially constructed (Figure S1). According to the number of AP2 domains contained, AP2/ERF family genes can be usually divided into ERF, AP2 and RAV subfamilies [4]. Furthermore, we found that both *Arabidopsis AINTEGUMENTA* (*ANT*) [26] and rice *qHD5* [17], containing two AP2 domains, were grouped into the AP2 subfamily. In this study, we specially focused on the pecan AP2 subfamily genes and further checked the candidate sequences through Smart (http://smart.embl-heidelberg.de, accessed on 12 July 2021). Interestingly, although *OF21942-RA*, *OF26251-RA*, *OF27743-RA* and *OF31010-RA* were clustered into the AP2 subfamily (Figure S1), they do not contain two AP2 domains. In this study, we preferentially focused on members containing two AP2 domains, and finally obtained 30 AP2 subfamily genes from pecan renamed from *CiAP2-1* to *CiAP2-7* and *CiANT1* to *CiANT23*, depending on their structural characteristics (Table 1). Furthermore, we analyzed the genomic distribution of AP2 genes, and results demonstrated that *CiAP2* genes were mainly distributed on chromosome 1, 6, 8 and 13 (Figure 1). Unfortunately, *CiANT13*, *16*, *19*, *20* and *22* were not anchored to the corresponding chromosomes.

### 2.2. Phylogenetic Analysis of Pecan AP2 Subfamily Genes

**Figure 2:**
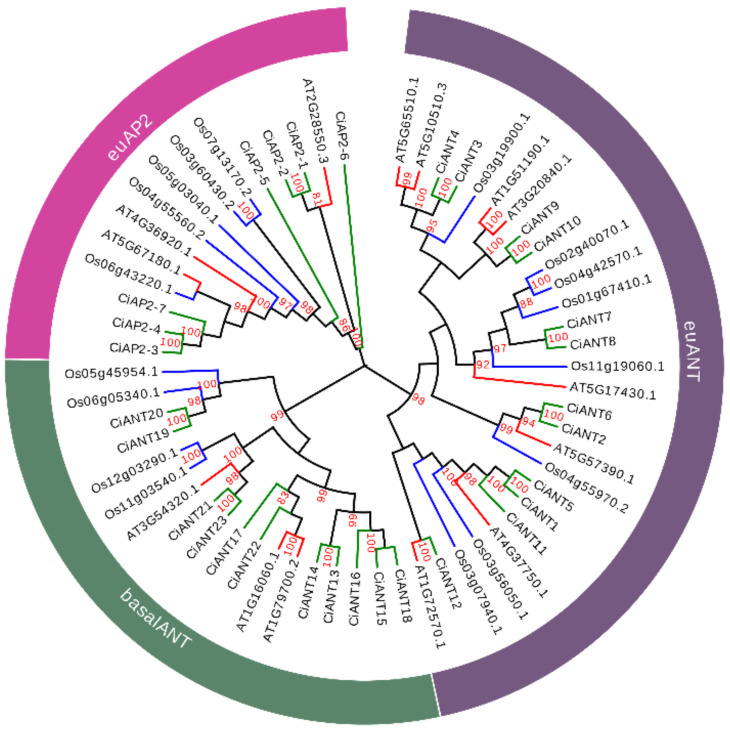
Phylogenetic analysis of AP2 subfamily proteins in the pecan, rice and *Arabidopsis*. A total of 61 AP2 proteins retrieved from pecan (30), rice (14) and *Arabidopsis* (17) were adopted to construct the phylogenetic tree. The tree was constructed using FastTree according to the maximum likelihood method. The AP2 subfamily proteins were clustered into three groups: euAP2, euANT and basal ANT. Proteins from pecan, rice and *Arabidopsis* are marked by green, blue and red colors, respectively.

To further reveal the evolutionary relationships among pecan AP2 subfamily proteins, we constructed a phylogenetic tree using the sequences of 30 pecan, 17 rice and 14 *Arabidopsis* AP2 subfamily proteins (Figure 2). The results were credible with high bootstrap values and demonstrated that pecan AP2 subfamily proteins could also be classified into the euAP2, euANT and basalANT three subgroups consistent with the previous study on AP2 family genes in *Arabidopsis* and rice [4]. We noted that *Arabidopsis ANT* (*AT4G37750*) and rice *qHD5* (*Os05g03040*) were clustered into euANT and euAP2, respectively. *ANT* is involved in ovule and female gametophyte development and controlled plant organ size by regulating growth and cell numbers during organogenesis in *Arabidopsis* [12,13]. *qHD5* was reported as a candidate gene for heading date in rice [17]. Therefore, although all AP2 subfamily genes contain two AP2 domains, they may regulate different biological processes.

### 2.3. Conserved Motif Analysis in AP2 Proteins

**Figure 3:**
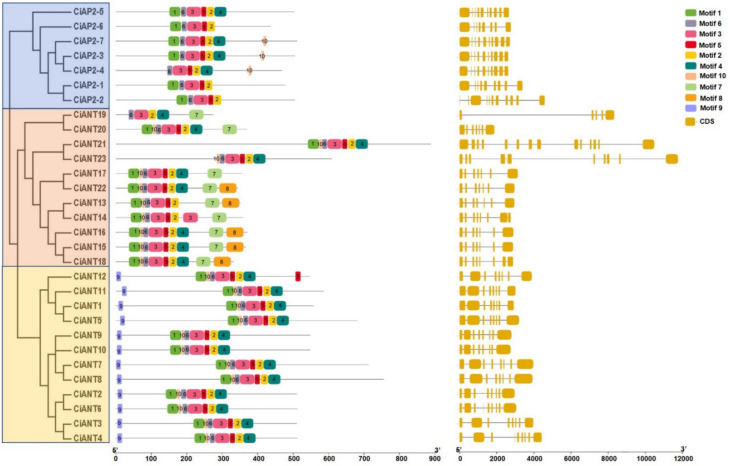
The motif distribution and gene structure of AP2 subfamily proteins in pecan. The middle panel represents the distribution of conserved motifs in CiAP2 proteins. The color blocks represent different motifs on proteins. The right panel represents the structure of CiAP2 genes. The green blocks represent exons.

The motifs in 30 AP2 protein sequences were analyzed through MEME. The results displayed the first ten conserved motifs, and they varied among euAP2, basalANT and euANT evolution branches (Figure 3). In euAP2, motifs 1, 2, 3, 4, 5 and 6 were found almost in all members. Among them, motif 6 was located in the first AP2 domain and motifs 1 and 3 were parts of the first AP2 domain. Motifs 2 and 4 were located in the second AP2 domain and motif 5 was part of the second AP2 between two AP2. Compared with the euAP2 group, basalANT and euANT contained the unique motif 10 in the first AP2 domain. BasalANT contained the unique motifs 7 and 8 on the C terminal, while euANT contained the unique motif 9 on the N terminal. Overall, the whole AP2 domain is presented in the middle of the euAP2 group, while near the N terminal in the basalANT group and in a diverse position in the euANT group. Moreover, the gene structures were visualized to further reveal the variations between *CiAP2* genes. As shown in Figure 3, the genes in the euANT group exhibited highly similar gene structures with 8 or 9 exons. Although the exon number ranged from 8 to 11 in euAP2 and 5 to 14 in the basalANT group, most genes showed similar gene structures in their respective groups except for *CiAP2-2* and *CiANT19*, *21*, *23*.

### 2.4. cis-Element Analysis of AP2 Subfamily Genes

**Figure 4:**
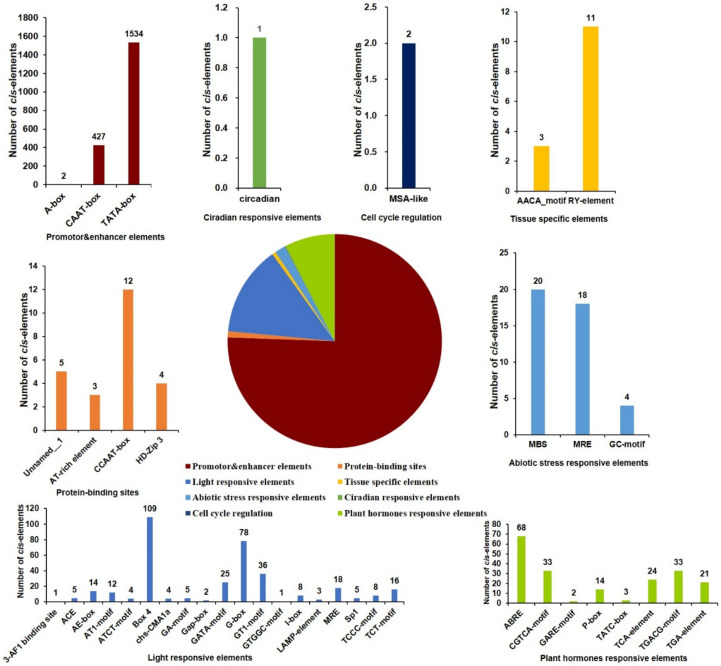
The analysis of *cis*-regulatory elements in the putative promoter of the AP2 subfamily genes. The pie chart shows the classification of *cis*-regulatory elements, and the corresponding color chart displays the frequency of different cis-elements.

AP2 family genes have been demonstrated to contribute to the processes of plant growth and development as well as various environmental stimuli. Therefore, detecting the characteristic of the *cis*-element in the promoter will deepen our understanding of the expression pattern of pecan AP2 genes. We analyzed the promoter through the PlantCARE server and clustered all motifs into eight groups depending on the function annotation (Figure 4). Evidently, promotor and enhancer elements accounted for the largest proportion, with TATA-box and CAAT-box occurring the most frequently. Light responsive elements and plant hormone-responsive elements ranked as the second and third largest proportion, respectively. Box4, G-box and GT1-motif were the top three in light responsive elements. We further predicted the plant hormone-responsive elements and found that abscisic acid, salicylic acid, MeJA, gibberellin and auxin responsive elements were identified in their promotors (Figure S2). Among them, the abscisic acid-responsive element ABRE and MeJA-responsiveness CGTCA-motif appeared the most frequently. We also found that abiotic stress responsive elements, such as the MYB binding site (MBS), were involved in drought inducibility, which implied *CiAP2* genes may participate in drought-stress response through the MYB transcription factor. Interestingly, we found tissue-specific elements were all *cis*-regulatory elements involved in seed-specific regulation, which was consistent with the function of AP2 genes participating in embryo development. Furthermore, we only identified one circadian-responsive and two-cell-cycle regulation elements in all AP2 subfamily genes. Overall, our results indicate that AP2 subfamily genes may participate in growth and development processes by responding to plant hormones, light and abiotic stress.

### 2.5. Expression Analysis of CiAP2 Genes

**Figure 5:**
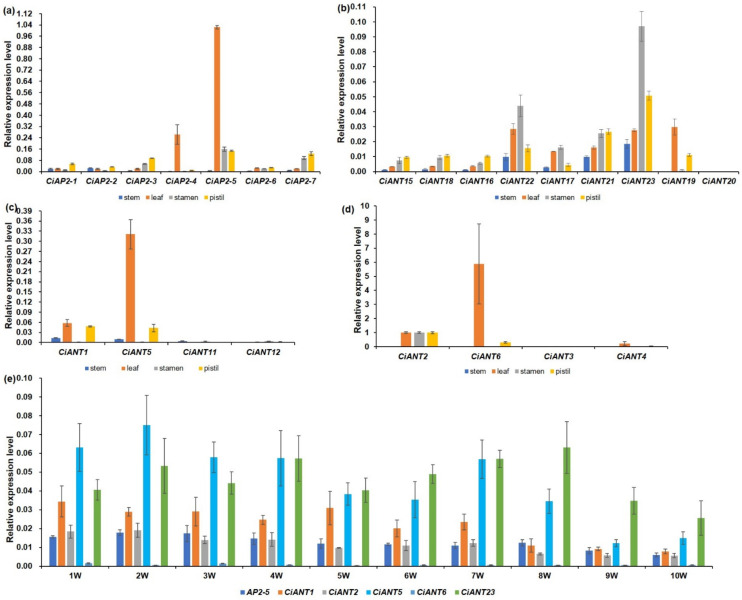
The expression analysis of pecan AP2 subfamily genes. (**a**–**d**) The expression of AP2 subfamily genes in pecan stems, leaves, stamens and pistils. (**e**) The expression of *CiAP2-5*, *CiANT1*, *CiANT5*, *CiANT6* and *CiANT23* in developing pecan fruits after pollened 1 to 10 weeks, respectively. Pecan *actin* gene (*OF23284*) was used as the internal reference gene. Error bars indicate SD. Each reaction represents three technical repeats.

To investigate the putative roles of pecan AP2 subfamily genes, a qPCR assay was employed to analyze their expression patterns in different tissues. Among the euAP2 clade, *CiAP2-5* had the highest relative expression level in leaves, stamens and pistils. The expression level of *CiAP2-7* was second in stamens and pistils. *CiAP2-1* and *CiAP2-2* were clustered together and presented consistent expression patterns (Figure 5a). Genes in basalANT clades, such as *CiANT15*, *16* and *18*, displayed similar expression patterns with relatively high expression levels in pistils, while *CiANT17*, *21*, *22* and *23* showed higher expression levels in stamens, especially *CiANT23* (Figure 5b). The expression pattern in the euANT clade varied greatly (Figure 5c,d). Although *CiANT1*, *5*, *11* and *12* were clustered together, *CiANT1* and *5* expressed higher, while *CiANT11* and *12* showed almost no expression in the tested tissues. The expression levels of *CiANT2* and *CiANT6* were higher in different tissues, while *CiANT3* displayed almost no expression in the tested tissues and *CiANT4* showed trace expression in leaves. Previous studies have demonstrated that some AP2 family genes participate in seed development in *Arabidopsis*. To identify the potential genes that are involved in pecan fruit development, we selected genes with a relatively high expression in the pistil for further quantitative analysis in the developing pecan fruits. As the results show (Figure 5e), *CiAP2-5*, *CiANT1* and *2* shared similar expression patterns in developing fruits, and their expression levels decreased with fruit development. The expression of *CiANT6* was lowest in developing fruits. *CiANT5* and *CiANT23* displayed the highest expression levels in developing fruits; *CiANT5* expressed higher in the first three weeks, while *CiANT23* expressed higher in the last three weeks during pecan fruit development. The difference in the expression levels implied that *CiANT5* and *CiANT23* were likely to function diversely during pecan fruit development.

### 2.6. CiANT5 Function as a Transcription Activator in Yeast

**Figure 6:**
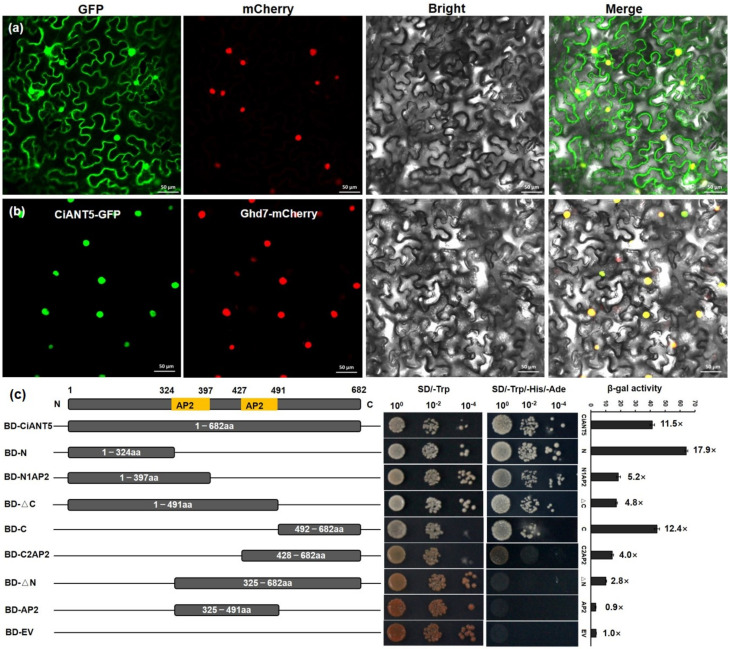
The subcellular and transcriptional analysis of CiANT5. (**a**) GFP fluorescence signal of GFP protein. (**b**) GFP fluorescence signal of the CiANT5-GFP fused protein. Ghd7-mCherry was used as nuclear maker. Bar = 50 μm. (**c**) The transcriptional activity analysis of CiANT5 in yeast *AH109* strain. The left diagram shows different constructions used for transcriptional assay. BD, GAL4-DNA binding domain; EV, empty *pGBKT7* vector used as a negative control. The middle panel displayed different constructions cultured on selective medium SD/-Trp and SD/-Trp/-His/-Ade. The right panel displays β-galactosidase (β-gal) activity in the various constructions. Mean values ± *SE* were calculated from three repeats.

As mentioned above, *CiANT5* demonstrated a high expression level in pecan leaves, pistil and developing fruits. Therefore, we selected it for further analysis. To explore whether CiANT5 is located in the nucleus as expected of a transcription factor, we conducted a subcellular localization analysis first. We obtained the full-length CDS of CiANT5 without a stop code through PCR amplification and fused CiANT5 to the N terminal of GFP. Then, the CiANT5-GFP and nuclear maker Ghd7-mCherry were transiently co-expressed in tobacco leaves. The results demonstrated that the CiANT5-GFP fluorescence signals localized in the nucleus overlapped perfectly with the red fluorescence of the nuclear maker Ghd7-mCherry, while free GFPs were detected throughout the nucleus and cytoplasm (Figure 6a,b). Subsequently, the transcriptional activation activity was analyzed in yeast cells. The full-length of CiANT5 as well as seven truncated fragments were ligated into the *pGBKT7* vector to generate BD-CiANT5(1-682aa), BD-N(1-324aa), BD-N1AP2(1-397aa), BD-∆C(1-491aa), BD-C(492-682aa), BD-C2AP2(428-682aa), BD-∆N(325-682aa) and BD-AP2(325-491aa) fusion constructions. These constructions as well as the empty *pGBKT7* were transformed into yeast *AH109* and cultured on selective medium SD/-Trp and SD/-Trp/-His/-Ade, respectively. As the result show, CiANT5 displayed transcriptional activation activity in yeast (Figure 6c). Among the six truncated fragments, except C2AP2, ∆N and AP2, others showed transcriptional activity. Furthermore, the results were confirmed by quantitatively measuring β-galactosidase (β-gal) activity. When the truncated fragments contained the AP2 domain, β-gal activity decreased and remained lower than the negative control in the BD-AP2 fragment. Taken together, these results demonstrate that CiANT5 is a transcription factor localized in the nucleus with transcriptional activity, while the AP2 domain has a negative effect on transcriptional activity.

## 3. Discussion

### 3.1. Characterization of Pecan AP2 Subfamily Genes

In this study, we initially identified 207 AP2/ ERF family genes in the pecan, which were classified into three subfamilies, ERF, RAV and AP2, depending on the number of AP2 domains (Figure S1). Of them, both ERF and RAV members contain a single AP2 domain, while members in the AP2 subfamily contain two tandem AP2 domains. Further, we verified the sequences and finally obtained 30 AP2 members with two AP2 domains, which was higher than that in *Pinus massoniana* (7) [27], Pineapple (24) [28], *Dendrobium officinale* (14) [24], while less than that in *Elaeis guineensis* (34) [29] and *Saccharum spontaneum* (43) [30]. Pecan AP2 members were also grouped into three clades, euAP2, euANT and basalANT, which is consistent with previous research reported in angiosperms, such as wheat and *Dendrobium officinale* [4,22,24]. Furthermore, the analysis of genomic distribution displayed that most genes were distributed on chromosomes 1, 6, 8 and 13 (Figure 1). Unfortunately, five genes in the basalANT clade, namely *CiANT13*, *16*, *19*, *20* and *22*, failed to be anchored on corresponding chromosomes. Similarly, three genes were not mapped to any chromosomes in pineapple [28], perhaps due to the imprecise chromosome assembly.

Proteins in the same family usually share a common characterized domain; however, members may contain diverse domains and motifs contributing to a variety of functions. A motif analysis showed that the top ten conserved motifs in the pecan AP2 subfamily TFs, and motifs 1, 2, 3, 4, 5 and 6 were found in almost all members (Figure 3), while motifs 7 and 8 were specific to basalANT and motif 9 was specific to euANT. This difference may lead to a diversity of gene functions. As reported, *miR172* binding to specific motif sites regulates euAP2 TF expression through transcript cleavage and translational repression [31,32]. In *Arabidopsis*, AP2 family members, such as *AP2*, *TOE1*, *TOE2*, *TOE3*, *SMZ* and *SNZ*, were *miR172* target genes. AP2 participated in floral organ formation, while others mainly suppressed floral formation [31,33]. Moreover, the gene structure reflected gene function and evolution to some extent. Our results show that the euANT group exhibited a highly similar gene structure with 8 or 9 exons, and the basalANT group had more diversified structures with 5 to 14 exons, which is consistent with previous findings in wheat [23]. In short, our results implied that different conserved motifs and gene structures may result in a diversity of functions responding to evolutionary selection pressures.

### 3.2. Promoter cis-Acting Elements and Expression Analysis Implied the Functions of Pecan AP2 TFs

AP2/ERF, as a plant-specific TF family, has been proven to play important roles during plant growth, abiotic stress and hormone response [2,34]. As expected, we found many tissue-specific elements, abiotic stress-responsive elements and plant hormone-responsive elements in the promoters of AP2 family genes (Figure 4). The tissue-specific elements mainly are the RY-element and AACA_motif involving seed and endosperm-specific expression regulation. Previous studies have demonstrated that petunia AP2-like genes PhAp2A, PhAp2B and PhAp2C are strongly expressed in endosperm and function in flower and seed development [35]. Wang et al. identified 57 putative TFs during maize seed development by expressed sequence tag (EST) analysis and found that three AP2/ERF members, ZmAP2-*1*, ZmAP2-*2* and ZmAP2-*2,* played important regulating roles in maize seed development [36]. In *Brassica napus*, *BABY BOOM* (*BBM*) belongs to the AP2/ERF family, preferentially expressed in developing embryos and seeds, and participates in cell proliferation and morphogenesis during embryogenesis [37]. Our results demonstrate that *CiAP2-5*, *CiANT1*, *2*, *5* and *23* displayed relatively higher expression in pistils and developing pecan fruits (Figure 5); they may participate in pecan fruit development. Besides, abiotic stress-responsive elements are another common element in the AP2 gene promoters, which may be related to the AP2 members regulating drought, heat, salt and freezing. *Arabidopsis* DREB1A/CBF1, DREB1B/CBF2 and DREB1C/CBF3 and their orthologous genes *OsDREB1A* and *OsDREB1B* in rice were involved in a low-temperature and drought response [7,38,39,40]. Zeng et al. [24] also found that the expression of *DoAP2* genes was changed to different degrees by being treated with cold, PEG and NaCl. Furthermore, we found that many elements respond to hormones in the promoters of pecan AP2 genes, of which ABA-responsive elements (ABRE) were the largest group (Figure 4 and Figure S2). This implied that the expression of *CiAP2* genes may be induced by ABA and that they may participate in various stress regulatory networks. Interestingly, MeJA-responsive elements were the largest group in *DoAP2* promoters [24]. This difference may be related to the optimum growing environment required by different species.

### 3.3. CiANT5 as a Member of AP2 TFs Dispayed Transcription Activity

Transcription factors can bind to specific DNA sequences and activate or repress the transcription of corresponding genes in the nucleus. Nuclear localization is the basic feature of transcription factors. In this study, we selected a homologous gene of *Arabidopsis ANT*, *CiANT5*, to conduct a subcellular localization assay. As expected, CiANT5, in the manner of SNB [20] and DoAP2-1, DoAP2-2, DoAP2-6 and DoAP2-11 [24], was part of the AP2 family TFs located in the nucleus (Figure 6b). Furthermore, we analyzed the transcriptional activity and found ANT5 functioned as a transcriptional activator in yeast. A truncated fragment analysis revealed that the activating domain was located in BD-N(1-324aa) and BD-C(492-682aa) (Figure 6c). While the two tandem AP2 or respective domains showed no activating function, perhaps they acted as binding domains bound to specific DNA motifs. Similarly, APETALA2-like TF SNB presented transcriptional activity in yeast, but the activating domain was located in residues 278 to 324 [20]. The C-terminal region (107-318) of FZP is indispensable for transcriptional activation activity by LUC activity in rice protoplasts [19]. These results implied the activating domain may be located in a diverse position in different AP2 TFs. Moreover, some AP2 TFs displayed transcriptional inhibitory activity. *Arabidopsis* AP2 negatively regulated the homeotic gene *AGAMOUS*, which is involved in floral organ development [41]. DoAP2-1, DoAP2-2, DoAP2-6 and DoAP2-11 displayed transcriptional repression activity in tobacco leaves [24]. Whether transcriptional activation or repression, both are the precise regulation of TFs to its target genes. Thus, the related transcriptional characteristics of the remaining pecan AP2 TFs still need additional research.

## 4. Materials and Methods

### 4.1. Identification of AP2 Subfamily Genes in Pecan

The genome sequences of the pecan were derived from our re-sequenced data. The AP2 family sequences of *Arabidopsis* and rice were downloaded from Phytozome (https://phytozome.jgi.doe.gov/pz/portal.html, accessed on 25 March 2021). The Hidden Markov Model of AP2 was downloaded from Pfam (http://pfam.xfam.org/, accessed on 27 March 2021), according to the Pfam id (PF00847). The HMM search from the HMMER program (v3.2.1) was employed to ascertain the presence of the AP2 domain in these three species. Furthermore, all candidate sequences were checked by Smart (http://smart.embl-heidelberg.de, accessed on 12 July 2021) to remove sequences without two AP2 domains and redundant sequences. The amino acid sequences of AP2 subfamily members are listed in Supplementary File S1.

### 4.2. Phylogenetic Analysis and Chromosome Localization of AP2 Subfamily Genes

The sequences were aligned using MAFFT (v7.271) [42], and the evolutionary tree was obtained by FastTree (v2.1.8) [43]. Then, the evolutionary tree was added colourful visualization plots through the online website evolview (www.evolgenius.info/evolview, accessed on 10 August 2021) [44]. The position of pecan AP2 subfamily genes on chromosomes were visualized by a localization tool in tbtools [45].

### 4.3. Gene Structure and Conserved Motif Analysis

According to the annotation files, the coding sequences and corresponding genomic sequences were extracted from the pecan genome. The conserved motif structure of AP2 subfamily genes was found by MEME (version 5.0.2) [46]. Then, all data were input into tbtools to visualize the gene structure and conserved motif.

### 4.4. cis-Element Analysis

A total of 2.0 Kb upstream sequences from initiation codon were retrieved as promoter sequences, and then the cis-regulatory elements were predicted through the PlantCARE database (http://bioinformatics.psb.ugent.be/webtools/plantcare/html/, accessed on 22 August 2021). Furthermore, we clustered all motifs into different groups depending on the function annotation and counted the frequency distribution of promotor and enhancer elements, protein-binding sites, light responsive elements, tissue specific elements, abiotic stress responsive elements, circadian responsive elements, cell cycle regulation and plant hormone responsive elements.

### 4.5. Plant Material and Sample Collection

The pecan cultivar Western used as plant material was grown in Lin’an City (30° N, 119° E), Zhejiang Province, China. The leaves, stems, stamens and pistils were collected from Western in late April 2021, when the stigmas were acceptable. Moreover, we collected developing fruits one to ten weeks after controlling for pollination. All samples were immerged in liquid nitrogen immediately after collection and stored at −80 °C in a refrigerator for its use.

### 4.6. RNA Isolation and qPCR Analysis

The RNA was isolated from pecan samples (leave, stems, stamens and pistils, as well as developing fruits) with modified CTAB combining TRIzol reagent (Invitrogen, Grand Island, NY, USA), as we used previously in [47]. The cDNA was synthesized using the Prime-Script™ RT Reagent Kit (TaKaRa, Kyoto, Japan), according to the manufacturer’s instructions.

qPCR was performed on a Bio-Rad CFX 92 Touch device using SYBR Green PCR Kit (TaKaRa). The procedure was as follows: 95 °C, 90 s; 40 cycles of 95 °C, 5 s; 55 °C, 20 s; 72 °C, 10 s. The relative expression level was calculated using the 2^−^^ΔΔCt^ method. The pecan actin gene (OF23284) was used as the internal reference gene. All primers used for the qPCR analysis are listed in Supplementary Table S1.

### 4.7. Subcellular Localization Analysis

The full-length CDS of CiANT5 without a stop code was amplified and inserted into the vector pYBA1132 between the XbaI and HindIII sites to form the fusion construct CiANT5-GFP. Ghd7-mCherry was used as nuclear maker. Different combinations of fusion constructs were co-transformed in *N. benthamiana* leaves. The EGFP signals were detected with a laser scanning confocal microscope (LSM880, Karl Zeiss, Oberkochen, Germany) 48 h after transfection. The primers used in the analysis are listed in Supplementary Table S1.

### 4.8. Transcriptional Activity Analysis

The transcriptional activity assay was performed using the Matchmaker GAL4 Yeast Two-Hybrid System 3 (Clontech, CA, USA). The full-length CDS and seven CiANT5 cDNA fragments were amplified and fused with pGBKT7 plasmid linearized by EcoRI. Empty pGBKT7 and fused plasmids, which were marked as BD-ANT5, BD-N, BD-N1AP2, BD-ΔC, BD-C, BD-C2AP2, BD-ΔN and BD-AP2 were transformed into the yeast strain AH109. All transformants were grown in a tryptophan-negative dropout medium (SD/-Trp), then incubated in selective media plates (SD/-Trp/-His/-Ade) at 30 °C for four days to assay transcriptional activity. Furthermore, chlorophenol red-β-D-galactopyranoside (CPRG, Roche, Basel, Switzerland) was used as a substrate to measure β-galactosidase activity via liquid culture assays, as we did previously in [48]. All the primers used for the vector construction are listed in Supplementary Table S1.

## Figures and Tables

**Table 1:** AP2 subfamily genes in Pecan.

No.	Gene ID	Gene Name	Gene Location	Predicted Protein Length (aa)	Isoelectric Point (pI)	Clade
1	OF00880-RA	CiAP2-1	Chr13: 16145978-16149358	476	8.28	euAP2
2	OF05517-RA	CiAP2-2	Chr16:22489793-22494373	504	8.82	
3	OF07993-RA	CiAP2-3	Chr01:62982646-62985262	504	7.43	
4	OF11627-RA	CiAP2-4	Chr01:65432895-65435508	468	6.46	
5	OF14885-RA	CiAP2-5	Chr05:11211192-11213844	502	7.26	
6	OF17041-RA	CiAP2-6	Chr01:1638337-1641093	436	7.10	
7	OF32680-RA	CiAP2-7	Chr14:27110789-27113491	510	7.08	
8	OF02559-RA	CiANT1	Chr06:6672431-6675340	556	6.85	euANT
9	OF02759-RA	CiANT2	Chr06:4414801-4417762	509	7.78	
10	OF03547-RA	CiANT3	Chr08:12993211-12997175	509	7.04	
11	OF09391-RA	CiANT4	Chr06:25934540-25938961	511	6.57	
12	OF10594-RA	CiANT5	Chr08:36743529-36746728	681	7.01	
13	OF10749-RA	CiANT6	Chr08:38789911-38792954	511	6.90	
14	OF13068-RA	CiANT7	Chr08:24346944-24350910	712	6.40	
15	OF18987-RA	CiANT8	Chr06:34173511-34177430	754	6.88	
16	OF22559-RA	CiANT9	Chr13:29470995-29473785	547	6.68	
17	OF22570-RA	CiANT10	Chr13:29876966-29879708	547	6.68	
18	OF32746-RA	CiANT11	Chr14:28189896-28192903	585	6.37	
19	OF32974-RA	CiANT12	Chr01:7995878-7999758	546	6.72	
20	OF08566-RA	CiANT13	ContigUN:49571770-49574715	350	8.28	basalANT
21	OF13593-RA	CiANT14	Chr11:34909386-34912135	358	7.64	
22	OF18890-RA	CiANT15	Chr02:51565314-51568192	366	7.39	
23	OF22034-RA	CiANT16	ContigUN:48433777-48436671	370	8.30	
24	OF24164-RA	CiANT17	Chr01:6433685-6436815	356	7.02	
25	OF24522-RA	CiANT18	Chr02:53441378-53444256	330	8.99	
26	OF28856-RA	CiANT19	ContigUN:3940772-3949092	274	6.26	
27	OF28858-RA	CiANT20	ContigUN:3974783-3976648	368	9.25	
28	OF29459-RA	CiANT21	Chr16:14006190-14016673	888	7.16	
29	OF30183-RA	CiANT22	ContigUN:15665370-15668325	343	8.06	
30	OF31987-RA	CiANT23	Chr13:7041337-7053094	608	4.68	

## Supplementary Materials

The following are available online at https://www.mdpi.com/article/10.3390/ijms222413568/s1: Figure S1: Phylogenetic analysis of AP2 superfamily proteins in pecan, rice and Arabidopsis; Figure S2: Prediction of hormone-responsive cis-elements in the putative promoters of CiAP2 genes; Table S1: Primers used in this study.
