# Supplementary figures and images for: Identification and Analysis of the AP2 Subfamily Transcription Factors in the Pecan (Carya illinoinensis)
Source: Int J Mol Sci. 2021 Dec 17;22(24):13568. doi: 10.3390/ijms222413568 (PMC8708044; doi:10.3390/ijms222413568)

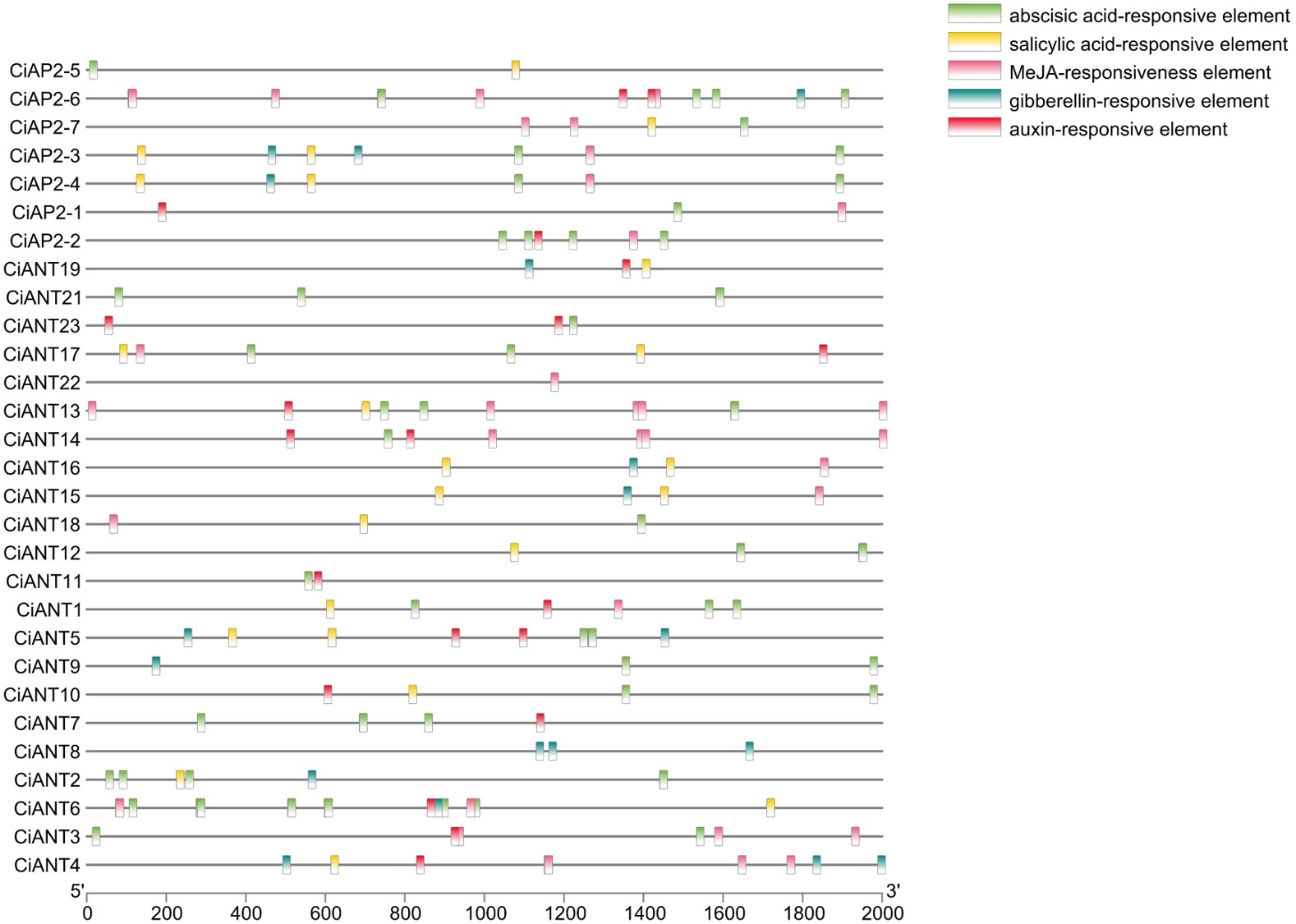

Supplement: Supplementary file 1 [file ijms-22-13568-s001.zip › Figure S2.jpg]
